# Tau‐induced nuclear envelope invagination causes a toxic accumulation of mRNA in *Drosophila*

**DOI:** 10.1111/acel.12847

**Published:** 2018-11-09

**Authors:** Garrett L. Cornelison, Simon A. Levy, Tyler Jenson, Bess Frost

**Affiliations:** ^1^ Department of Cell Systems and Anatomy, Barshop Institute for Longevity and Aging Studies, Glenn Biggs Institute for Alzheimer’s and Neurodegenerative Diseases University of Texas Health San Antonio San Antonio Texas

**Keywords:** nuclear export, nucleocytoplasmic transport, nucleus, RNA, tau, tauopathy

## Abstract

The nucleus is a spherical dual‐membrane bound organelle that encapsulates genomic DNA. In eukaryotes, messenger RNAs (mRNA) are transcribed in the nucleus and transported through nuclear pores into the cytoplasm for translation into protein. In certain cell types and pathological conditions, nuclei harbor tubular invaginations of the nuclear envelope known as the “nucleoplasmic reticulum.” Nucleoplasmic reticulum expansion has recently been established as a mediator of neurodegeneration in tauopathies, including Alzheimer's disease. While the presence of pore‐lined, cytoplasm‐filled, nuclear envelope invaginations has been proposed to facilitate the rapid export of RNAs from the nucleus to the cytoplasm, the functional significance of nuclear envelope invaginations in regard to RNA export in *any disorder* is currently unknown**.** Here, we report that polyadenylated RNAs accumulate within and adjacent to tau‐induced nuclear envelope invaginations in a *Drosophila* model of tauopathy. Genetic or pharmacologic inhibition of RNA export machinery reduces accumulation of polyadenylated RNA within and adjacent to nuclear envelope invaginations and reduces tau‐induced neuronal death. These data are the first to point toward a possible role for RNA export through nuclear envelope invaginations in the pathogenesis of a neurodegenerative disorder and suggest that nucleocytoplasmic transport machinery may serve as a possible novel class of therapeutic targets for the treatment of tauopathies.

## 1 INTRODUCTION, RESULTS, DISCUSSION

The nucleus is typically depicted as a smooth spherical structure bound by the inner and outer membranes of the nuclear envelope and supported by an interior filamentous protein meshwork known as the Lamin nucleoskeleton. In certain cell types and pathological conditions, such as cancer and Laminopathies, this smooth exterior is interrupted by invaginations of the nuclear envelope, which are referred to as a “nucleoplasmic reticulum” (Malhas, Goulbourne, & Vaux, 2011). When both inner and outer nuclear membranes invaginate, the nucleoplasmic reticulum is cytoplasm‐filled and lined with nuclear pores. While relatively little is known about nucleoplasmic reticulum function or the consequences of nucleoplasmic reticulum expansion in pathological states, it is thought that the nucleoplasmic reticulum may bring functions of the nuclear periphery, such as nuclear export of RNAs, into the nuclear interior (Frost, 2016; Malhas et al., 2011). Kinetic studies on the transport of single messenger ribonucleoproteins indicate that diffusion from the site of transcription to nuclear pore complexes within the nuclear envelope is the rate‐limiting step in mRNA export (Mor et al., 2010), suggesting that a decrease in diffusion distance mediated by nuclear envelope invagination could result in a net‐increase in the rate of mRNA export.

Tauopathies, including Alzheimer's disease, are neurodegenerative disorders that involve aberrant accumulation and deposition of tau protein within the brain (Arendt, Stieler, & Holzer, 2016). Tau‐induced reduction of Lamin protein levels has recently been reported to cause nucleoplasmic reticulum expansion in tau transgenic *Drosophil*a and human patients with Alzheimer's disease (Frost, Bardai, & Feany, 2016). Pan‐neuronal expression of a disease‐causing mutant form of human tau, tau^R406W^, resulted in nuclear invagination in approximately 15% of neurons in adult flies, with approximately 86% of these invaginated nuclei co‐localizing with disease‐associated epitopes of phosphorylated tau. Analysis of postmortem brain tissue from patients with Alzheimer's disease showed that approximately 60% of neurons contain nuclear envelope invaginations, and that these invaginations are lined with nuclear pores, raising the possibility that nucleoplasmic reticulum expansion could facilitate RNA export in the context of tauopathy. Here, we utilize tau^R406W^ *Drosophila* to investigate how tau‐induced nucleoplasmic reticulum expansion affects RNA export, and what role, if any, this plays in mediating tau‐induced neuronal death.

**Figure 1:**
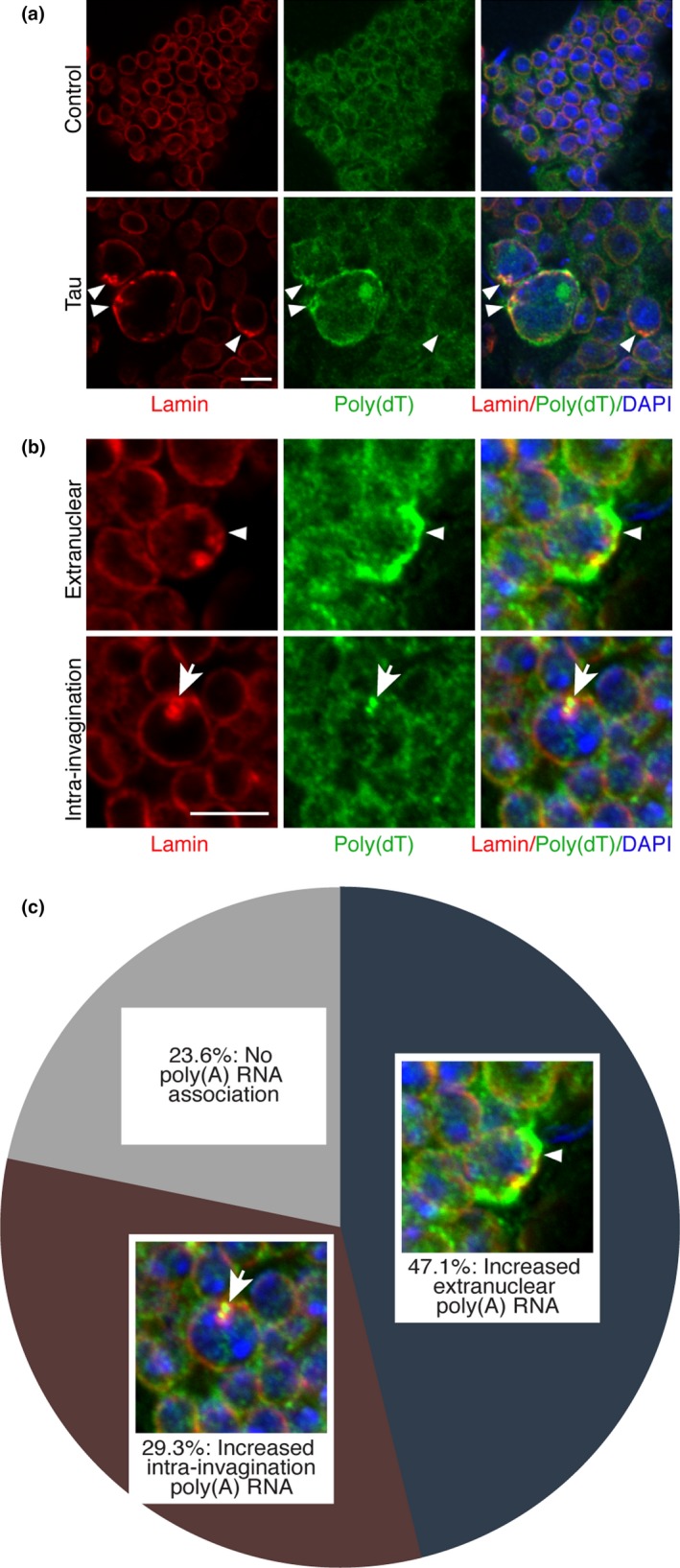
Polyadenylated RNA accumulates at nuclear invaginations in tau transgenic *Drosophila*. (a) Immunostaining of Lamin and fluorescence in situ hybridization of polyadenylated RNA with a poly(dT) nucleotide probe in control and tau transgenic *Drosophila* brains. Arrowhead indicates nuclear invaginations. (b) Comparison of extranuclear versus intra‐invagination accumulation of polyadenylated RNA in nuclei of tau transgenic *Drosophila* brains. Arrowhead indicates extranuclear accumulation of polyadenylated RNA, while arrow indicates intra‐invagination accumulation of polyadenylated RNA. (c) Pie chart depicting the percent of invaginated nuclei in tau transgenic *Drosophila* that are associated with either extranuclear, intra‐invagination, or no polyadenylated RNA accumulation, *n* = 4, 100 nuclei per fly. All flies are 10 days old. Control is *elav‐Gal4/+*. Scale bars represent 5 μm

To investigate whether tau‐induced nucleoplasmic reticulum expansion affects mRNA export, we combined fluorescence in situ hybridization with immunofluorescence (FISH‐IF) using a poly(dT) nucleotide probe to visualize polyadenylated RNA and an antibody recognizing Lamin to visualize nuclear architecture. While control flies have relatively consistent levels of RNA between cells, tau transgenic flies show a marked accumulation of polyadenylated RNA associated with invaginated nuclei (Figure 1a, staining with poly(dA) control probe presented in Supporting information Figure S1). The majority of nuclei harboring an invagination in tau transgenic flies display increased RNA staining localized within the nuclear invagination itself, or spread along the cytoplasmic edge of the nucleus, adjacent to the invagination (Figure 1b,c), consistent with a putative role for a nucleoplasmic reticulum‐mediated RNA export. We also observed that tau transgenic flies contain many nuclei that are larger than those found in controls, in line with previous observations of increased nuclear size as a result of nucleoplasmic reticulum expansion (Saltel et al., 2017).

**Figure 2:**
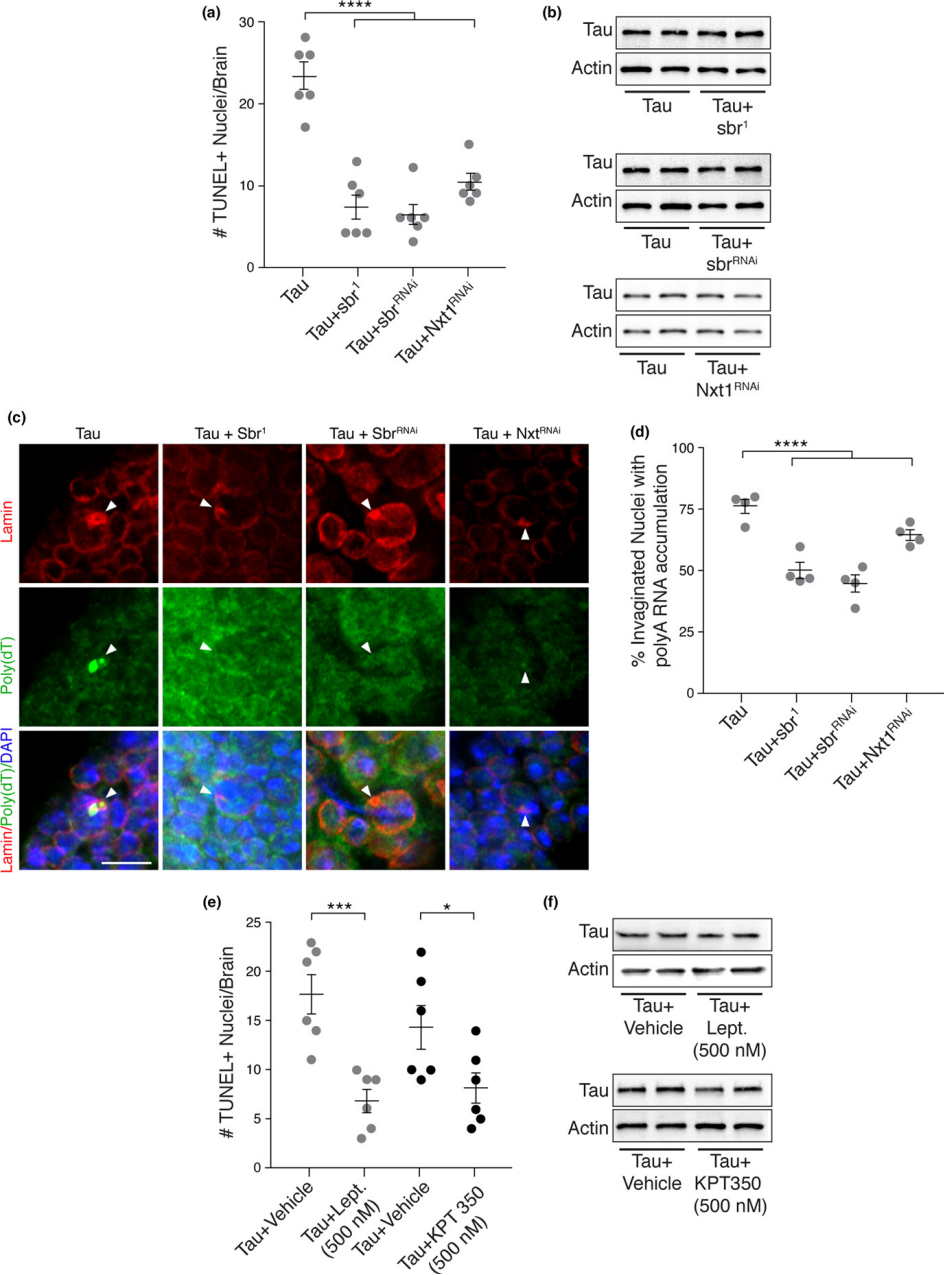
Genetic or pharmacologic inhibition of RNA export machinery reduces tau‐induced toxicity in vivo. (a) Neuronal degeneration assayed by TUNEL staining; *n* = 6. (b) Western blot showing human tau protein levels in *Drosophila* heads. Representative images (c) and quantification (d) of the fraction of nuclear invaginations associated with RNA accumulation in *Drosophila* brains harboring the indicated allele or transgene; *n* = 4, 100 nuclei per fly. (e) Neuronal degeneration assayed by TUNEL staining in tau transgenic *Drosophila* treated with either 500 nM leptomycin B (Lept.), 500 nM KPT 350, or the appropriate vehicle. (f) Western blot showing tau protein levels in tau transgenic *Drosophila* treated with leptomycin B (Lept.), KPT350, or the appropriate vehicle. Arrowheads indicate Lamin invaginations. All flies are 10 days old. Controls are *elav‐Gal4*/+. Data are presented as the mean ± sem; unpaired *t* test or ANOVA with Dunnett's post hoc test, *p < 0.05; ***p < 0.001; ****p < 0.0001

To determine if aberrant mRNA accumulation is a result of increased RNA export and contributes to tau‐induced neurotoxicity, we examined the effects of genetic or pharmacologic inhibition of RNA export machinery on tau‐induced neuronal death. *Nxt1* and *sbr* are *Drosophila* homologs of human nuclear transport factor 2‐like export factor 1, *NXT1*, and nuclear RNA export factor 1, *NXF1*, which encode proteins that are critical for nuclear pore‐mediated RNA export (Sloan et al., 2016). Tau transgenic *Drosophila* harboring a loss‐of‐function mutation in *sbr* (*sbr^1^*), or RNAi‐mediated knockdown of sbr or Nxt1 have significantly less neuronal death compared to tau expressed alone based on terminal deoxynucleotidyl transferase dUTP Nick‐End Labeling (TUNEL) (Figure 2a). Importantly, these genetic manipulations do not affect protein levels of the human tau transgene (Figure 2b). Additionally, genetic reduction of RNA export in tau transgenic *Drosophila* significantly reduces the number of invaginations associated with increased RNA staining in tau transgenic flies (Figure 2c,d). Similar to genetic inhibition of RNA export, treatment of tau transgenic *Drosophila* with 500 nM leptomycin B or KPT‐350, selective inhibitors of Exportin‐1 (XpoI)‐mediated nuclear transport (Haines et al., 2015), throughout adulthood also results in significantly less neuronal death in tau transgenic *Drosophila* compared to vehicle‐treated animals, without changes in transgenic tau protein levels (Figure 2e,f).

We have previously shown that pathogenic tau reduces total Lamin protein levels, which is causally linked to nuclear envelope invagination and subsequent neuronal death (Frost et al., 2016). Indeed, TUNEL‐positive nuclei in tau transgenic *Drosophila* harbor both nuclear envelope invaginations and contain very little Lamin protein compared to control (Supporting information Figure S2a). To determine the direct contribution of reduced Lamin function on RNA accumulation, we performed FISH‐IF in brains of *Drosophila* homozygous for a partial loss‐of‐function allele of *Lamin*,* Lam^A25^*. *Drosophila* harboring the homozygous *Lam^A25^* allele share critical cellular phenotypes with tauopathies, including heterochromatin decondensation, DNA damage, and neuronal death (Frost et al., 2016). Similarly, we find widespread accumulation of polyadenylated RNA in brains of *Lam^A25^* homozygotes compared to controls (Supporting information Figure S2b), suggesting that tau‐induced Lamin dysfunction and subsequent nuclear envelope invagination are mechanistic drivers of the aberrant RNA trafficking and localization in tau transgenic *Drosophila*.

Recently, disruption of nuclear architecture and nucleocytoplasmic transport have been reported in the context of *C9orf72* repeat expansion and TAR DNA‐binding protein‐43 (TDP‐43) mediated neuronal death, common among patients with amyotrophic lateral sclerosis and frontotemporal dementia, as well as in Huntington's disease (Chou et al., 2018; Freibaum et al., 2015; Gasset‐Rosa et al., 2017). However, these studies uniformly report deficits in RNA export characterized by retention of RNAs within the nucleus. Additionally, contrary to our current findings, genetic reduction of sbr and XpoI *enhances* toxicity in a *Drosophila* model of *C9orf72* repeat expansion (Freibaum et al., 2015). In the context of previous studies of RNA export in non‐tau mediated neurodegeneration, our current findings suggest that the nature of nucleocytoplasmic transport dysfunction is distinct between tauopathies and other neurodegenerative disorders.

It is currently unclear why a potential increase in RNA export would be toxic to neurons. Based on the widespread decondensation of transcriptionally silencing heterochromatin in tauopathies (Frost, Hemberg, Lewis, & Feany, 2014), it is possible that RNA export‐mediated toxicity occurs through the production of transcripts that are normally absent in brains unaffected by tauopathy. Alternatively, an increased rate of RNA export may circumvent or overwhelm RNA quality control mechanisms, allowing export of nonfunctional RNAs and production of abnormal peptides that naturally and frequently occur due to errors in transcription (Hug, Longman, & Cáceres, 2016). Similarly, an increased transcript load could overload the translational capabilities of a cell, throwing off balance the dynamic homeostasis of regulated protein production required by healthy cells. While sbr and Nxt1 are primarily involved in mRNA export, XpoI is known to play a larger role in both the import and export of various proteins and noncoding RNAs (Sloan et al., 2016), suggesting that aberrant nucleocytoplasmic transport of multiple RNA species or proteins may also mediate tau‐induced neurotoxicity. While our findings identify nucleocytoplasmic transport machinery as a class of novel putative therapeutic targets for the treatment of tauopathies that warrant further investigation, we cannot exclude the possibility that sbr and Nxt1 have functions outside of RNA export that explain why their decreased function suppresses tau‐induced neuronal death.

We report that polyA RNA accumulates within nuclear envelope invaginations and that genetic knockdown of RNA export machinery suppresses tau‐induced neurotoxicity. In our view, the simplest interpretation of these data is that tau‐induced nuclear envelope invaginations facilitate a toxic increase in RNA export. We cannot yet claim, however, that nuclear envelope invaginations directly mediate RNA export. It is possible that polyA RNAs simply become “stuck” in invaginations due to the physical barrier of the invagination itself, or that RNAs within invaginations fail to untether from nuclear pores. Future studies are required to test these models and to determine whether our studies are relevant to vertebrate tauopathy.

## 2 EXPERIMENTAL PROCEDURES

### 2.1 Genetics and animal models

All *Drosophila melanogaster* stocks and crosses were maintained at 25°C under a 12‐hr light/dark cycle. All experimental procedures and analyses were performed on flies at day 10 of adulthood (day 10 post‐eclosion). Neuronal expression of RNAi and transgenes in *Drosophila* was achieved using the *Gal4/UAS* system. GAL4 expression was driven by the pan‐neuronal *elav* promoter in all experiments. The *UAS*‐Tau^R406W^ line was a gift from Mel Feany and has been previously described (Wittmann et al., 2001). *W^1118^* (line number 3605), *sbr^1^* (line number 94) and *Lam^A25^* (line number 25092) lines were obtained from the Bloomington Drosophila stock center. The *sbr^RNAi^* (line number 103715) and *Nxt1^RNAi^*(line number 52631) lines were obtained from the Vienna *Drosophila* Resource Center.

### 2.2 Co‐fluorescent in situ hybridization/immunofluorescence (FISH‐IF).

Formalin‐fixed, paraffin‐embedded *Drosophila* heads were sectioned at 4 μm and subjected to 20‐min pressure cooker‐based antigen retrieval in 10 mM sodium citrate (pH 6.6) with 0.2% tween. Sections were incubated with 1 ng/ml of either 5′3′ DIG‐labeled Poly(dT24) or Poly(dA24) probes in hybridization buffer consisting of 2× SSC (300 mM NaCl, 30 mM sodium citrate, pH 7.0) with 20% formamide and 10% dextran sulfate at 37°C for 4 hr followed by three 10 min washes in 2× SSC at 37°C. Sections were briefly rinsed with PBS + 0.2% Triton‐X 100 (PBSTr), blocked for 30 min in PBSTr + 2% milk, and incubated with 1:100 α‐Lamin (ADL67.10; DSHB, Iowa City, IA) and 1:200 α‐DIG (R&D Systems, Minneapolis, MN) antibodies overnight at room temperature. Secondary detection was performed using Alexa Fluor^TM^‐conjugated secondary antibodies (ThermoFisher Scientific, Waltham, MA, USA). All images were taken with a Zeiss LSM 780 upright confocal microscope and analyzed with ImageJ software. All images shown are single slice. In comparing control versus tau transgenic flies, direct observational comparisons were made from samples that were processed identically and at the same time. Polyadenylated RNA accumulation was scored as an area where there was an obvious increase in poly(dT) probe signal compared to the surrounding cells in the sample. Intra‐invagination accumulations of polyadenylated RNA were counted as accumulations that were obviously contained completely within an invagination, as imaged with Lamin staining, and extranuclear accumulations were counted as areas where RNA was obviously present on the cytoplasmic side of an invagination. Accumulation was considered extranuclear even if poly(dT) signal was also contained within the invaginations themselves.

### 2.3 Drug treatments

Leptomycin B (Cayman Chemical) was dissolved in 100% ethanol. KPT 350 was a gift from Karyopharm Therapeutics (Newton, MA, USA) and dissolved in DMSO. On the day of eclosion (day 0), flies were transferred to food containing 500 nM of drug or the appropriate vehicle. Flies were flipped to fresh vials of drug or vehicle‐treated food twice a week. Ten days post‐eclosion, flies were collected and fixed in formalin for TUNEL staining or frozen at −80°C for Western blotting.

### 2.4 Western blotting

Frozen *Drosophila* heads were homogenized in 20 μl 2× Laemmli sample buffer (Sigma‐Aldrich, St. Lois, MO, USA), boiled at 100°C for 10 min, run on 15% SDS‐Page precast gels (Bio‐Rad Laboratories, Hercules, CA, USA) and transferred to nitrocellulose membranes following standard procedures. Equal loading and transfer efficiency were assessed by Ponceau S staining (Sigma‐Aldrich, St. Lois, MO, USA). Membranes were blocked in blocking buffer (PBS, 0.1% tween, and 2% milk) for 5 min followed by overnight incubations with the appropriate primary antibodies diluted in blocking buffer at 4°C. Primary antibodies were Tau (DAKO, A0024), actin (Abcam, ab8227). The next day, membranes were washed three times for 5 min in PBS + 0.1% tween followed by a 2‐hr incubation with HRP‐conjugated secondary antibodies at room temperature. Blots were then washed twice for 5 min with PBS + 0.1% tween, once for 5 min with dH2O and developed with an enhanced chemiluminescent substrate (ThermoFisher Scientific).

### 2.5 Immunohistochemistry and immunofluorescence

Neuronal cell death was assayed by TUNEL staining (TdT FragEL Kit; Calbiochem, San Diego, CA, USA) in 4 μm thick sections prepared from formalin‐fixed, paraffin‐embedded *Drosophila* heads. Colorimetric detection of TUNEL labeled nuclei was performed using the VECTASTAIN Elite ABC Kit and DAB substrate (Vector Laboratories, Burlingame, CA, USA). TUNEL labeled nuclei were counted throughout the entire brain. For Lamin/TUNEL co‐staining, *Drosophila* brains were dissected in PBS and fixed for 20 min in methanol prior to staining. Primary antibodies were anti‐Lamin Dm0 (DSHB, ADL67.10) and anti‐DIG (R&D Systems, MAP7520). Secondary detection was performed using Alexa Fluor^TM^‐conjugated secondary antibodies and Alexa Fluor^TM^‐conjugated streptavidin (ThermoFisher Scientific) diluted in PBSTr + 2% milk. All images were taken with a Zeiss LSM 780 upright confocal microscope and analyzed with ImageJ software. All images shown are a single slice.

### 2.6 Statistical analyses

All statistical data analysis was performed with Prism 7 (GraphPad Software, Inc., La Jolla, CA). Statistical analyses were performed using unpaired student's *t* test when comparing two variables or one‐way ANOVA with Dunnett's post hoc test when making multiple comparisons, as indicated.

## CONFLICT OF INTEREST

None.

## AUTHOR CONTRIBUTIONS

GLC, SAL and TJ performed experiments. GLC performed statistical analyses. GLC, SAL and BF designed the study and prepared the manuscript.
