# Supplementary figures and images for: Tau‐induced nuclear envelope invagination causes a toxic accumulation of mRNA in Drosophila
Source: Aging Cell. 2018 Nov 9;18(1):e12847. doi: 10.1111/acel.12847 (PMC6351838; doi:10.1111/acel.12847)

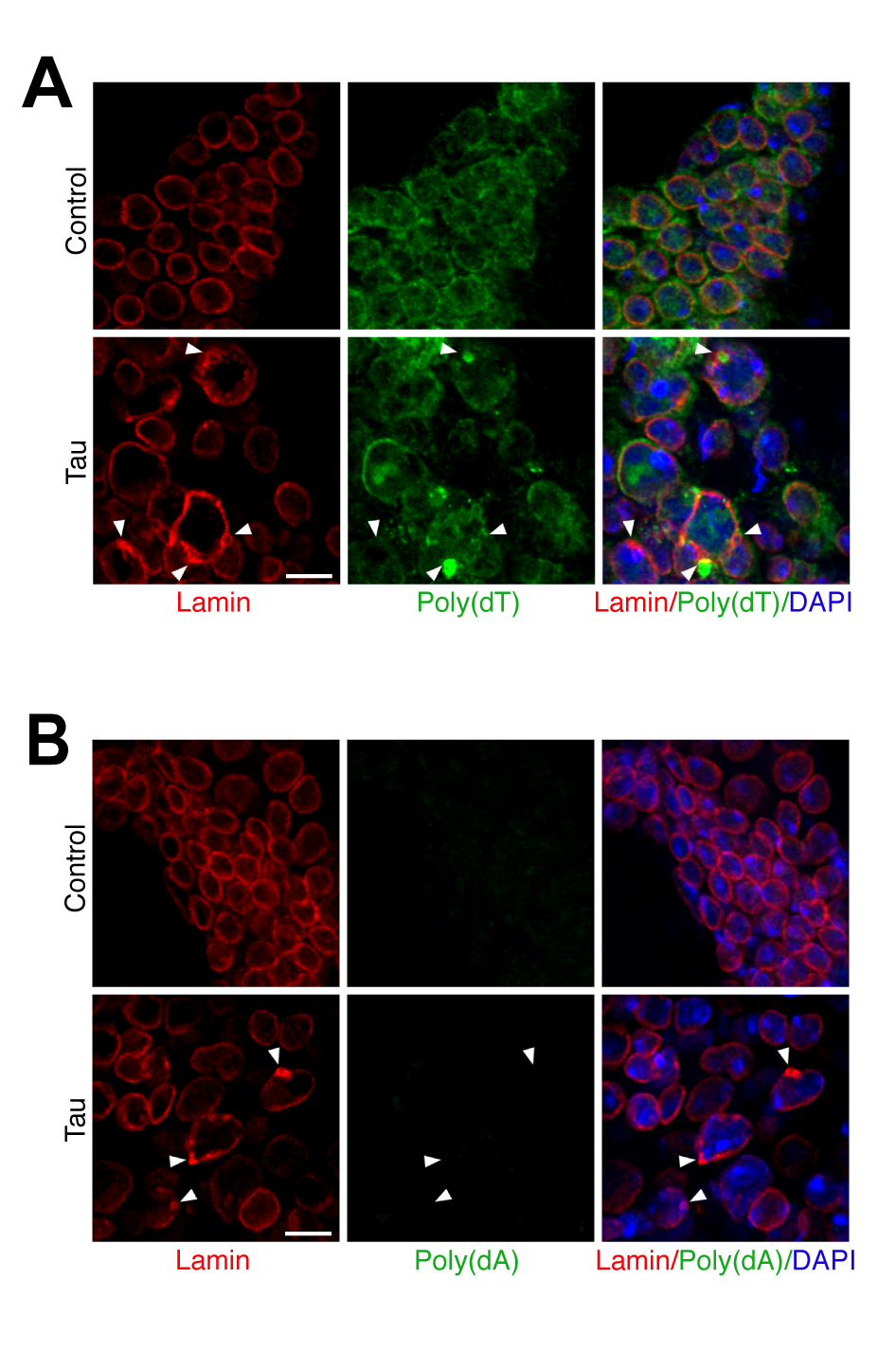

Supplement: Supplementary file 1 [file ACEL-18-e12847-s001.tif]

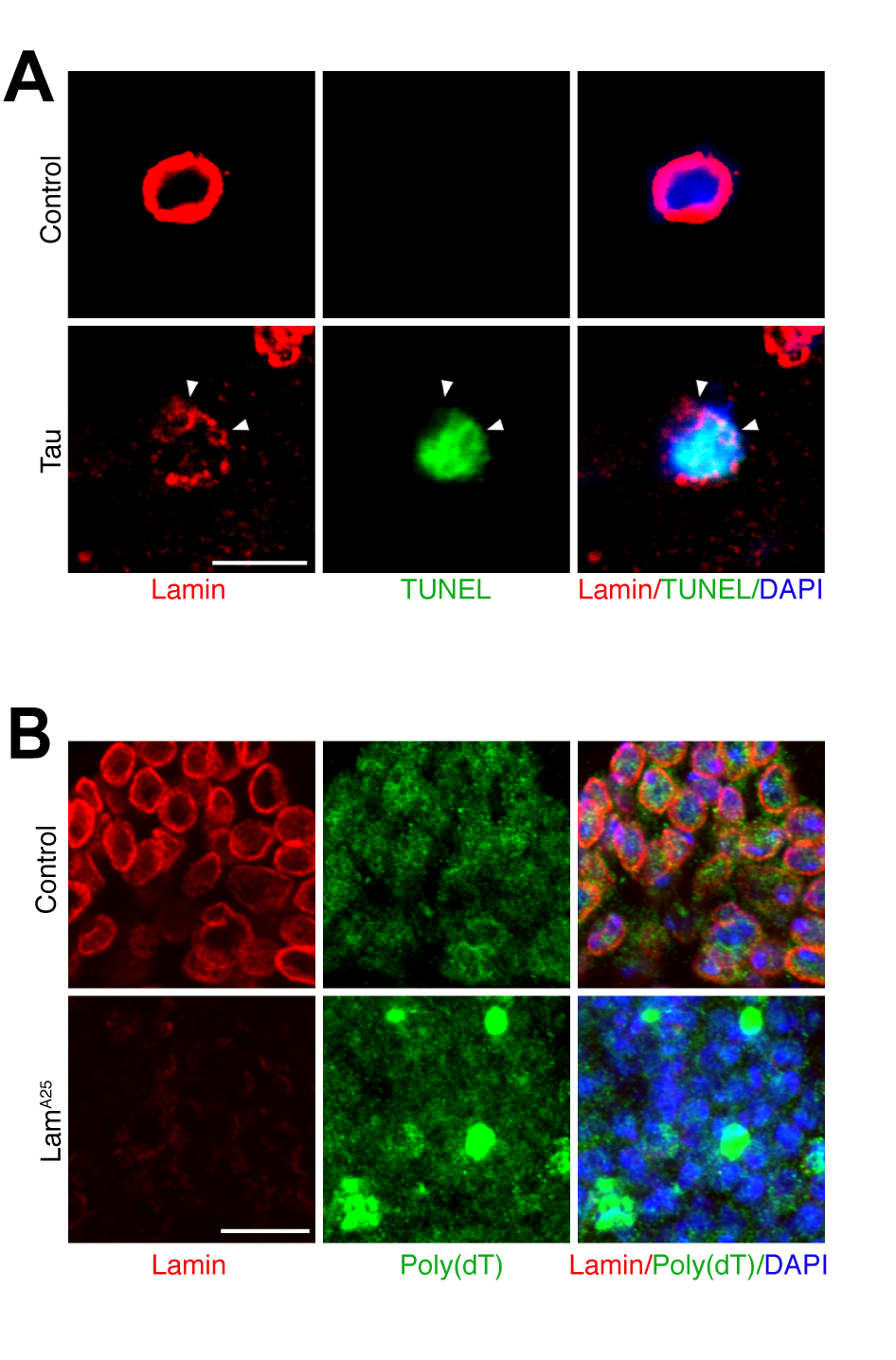

Supplement: Supplementary file 2 [file ACEL-18-e12847-s002.tif]
